# Enhancement of Localization Systems in NLOS Urban Scenario with Multipath Ray Tracing Fingerprints and Machine Learning †

**DOI:** 10.3390/s18114073

**Published:** 2018-11-21

**Authors:** Marcelo N. de Sousa, Reiner S. Thomä

**Affiliations:** Institute for Information Technology, Technische Universität Ilmenau, P.O. Box 100565, D-98684 Ilmenau, Germany; reiner.thomae@tu-ilmenau.de

**Keywords:** wireless positioning, cooperative positioning, machine learning, hybrid positioning, multipath exploitation, time difference of arrival localization, ray tracing fingerprints

## Abstract

A hybrid technique is proposed to enhance the localization performance of a time difference of arrival (TDOA) deployed in non-line-of-sight (NLOS) suburban scenario. The idea was to use Machine Learning framework on the dataset, produced by the ray tracing simulation, and the Channel Impulse Response estimation from the real signal received by each sensor. Conventional localization techniques mitigate errors trying to avoid NLOS measurements in processing emitter position, while the proposed method uses the multipath fingerprint information produced by ray tracing (RT) simulation together with calibration emitters to refine a Machine Learning engine, which gives an extra layer of information to improve the emitter position estimation. The ray-tracing fingerprints perform the target localization embedding all the reflection and diffraction in the propagation scenario. A validation campaign was performed and showed the feasibility of the proposed method, provided that the buildings can be appropriately included in the scenario description.

## 1. Introduction

We propose an approach to enhance TDOA localization Systems in urban scenario binding together a ray-tracing propagation tool to extract the multipath fingerprint and a machine learning framework to improve the precision even in NLOS situation. Contrary to similar approaches [1], where the NLOS measurements are not used in the position estimation, we build a multipath exploitation scheme that uses ray tracing and machine learning to give an extra layer of information using the buildings and obstacles in the environment. Despite the limitation of the primary results, in some scenarios, where the obstacles do not change their position, the channel impulse response estimation can be improved by repeatedly averaging the delays and power of the multipath components.

The localization of an electromagnetic source using a set of geographic spatially separated sensors is an essential problem in radar, sonar, and global positioning systems and mobile communications. The position of a target of interest can be determined by utilizing its emitted signal measured at an array of spatially separated receivers, also known as “observers” or sensors, with a priori known locations.

Among several parameters that can be measured from the received signal, time of arrival (TOA), time difference of arrival (TDOA), received signal strength (RSS), and direction of arrival (DOA), of the emitted signal, are the most commonly used for source localization. TOAs, TDOAs, and RSSs provide the distance information between the source and sensors, while DOAs are the source bearings relative to the receivers [2].

The emitter position can be estimated using the range between the source and observer taken from the TOAs, TDOAs, and RSS measurements. However, the distance and position have a nonlinear relationship. Another drawback effect in range-based systems is the multipath propagation caused by the obstacles in the scenario, where a non-line-of-sight (NLOS) ray can cause large positive biases in the estimated distance. The main algorithms to perform position estimation are the nonlinear least squares (NLS) and maximum likelihood (ML), which try to use the nonlinear relationships between the source and measurements. There are also linear least squares (LLS), weighted linear least squares (WLLS), and subspace approaches, which are based on the linearization in the range-difference function. It is also possible to try to model the positioning as an unconstrained optimization problem, using expressions for the mean and variance of the error as cost functions [3].

The NLOS propagation takes place when there are obstructions (e.g., walls, vegetation, buildings, and mountains) between the transmitter (TX) and the receiver (RX). The conventional TDOA methods try to take into account only line-of-sight (LOS) measurements and discard the NLOS paths in the position estimation. Multipath is an intrinsic urban channel impulse characteristic that is difficult to eliminate. In practical outdoor scenarios, the availability of positions to deploy sensors are scarce.

Multipath is typically perceived as a limitation but is possible to exploit interaction effects for localization of the RF emitter using a single sensor. There are various approaches [4] for tracking and vehicle position finding where multipath exploitation is performed to locate the target, again using the image-based approach to identify the sensor position end extract the location with LOS and NLOS calculation. In [5], the multipath information is used together with the image theory to locate either an emitter or a target, showing the feasibility to perform location finding using only one sensor. When considering electromagnetic sensors, the performance of the system not only depends on the localization algorithms or any other techniques since it is also disturbed by the multipath where the NLOS rays affect the time measurements.

This approach is relevant to enhance radio-frequency localization performance in an urban environment because it uses NLOS and geographic database as an extra layer of information, which can be fused in the position estimation engine. The idea was to apply such a method when the emitter is in a severe multipath situation, which would give a refinement in the position estimation done for RF sensor systems. There is always a trade-off between the performance desired and the available resources to deploy the number of sensors needed. For this reason, it is essential to improve the performance of available sensors even in NLOS condition, trying to extract as much information as possible from the deployed environment.

### 1.1. Review of Passive Emitter Localization Techniques

There is extensive literature on localizing 3D emitter based on measurements collected at sensors which are spatially distributed. The sensor measurements TOA, TDOA, FDOA, and DOA are considered in this case. On the other hand, more complicated and hybrid sensors make the localization approach more effective. Among the single sensor approaches, hybrid or mixed sensors have been considered and show good results for emitter position estimation. One of them is DOA combined with RSS [6,7]. Compared with DOA, RSS localization is less accurate. The combined approach in this paper improves the localization accuracy. TDOA and DOA measurements are considered in [8,9] and also investigated in two dimensional (2D) space. The hybrid sensor’s result is two-fold. Some recent work on hybrid localization for TDOA and DOA is illustrated in [10] and TDOA and TOA are combined and described under 3D configuration in [11,12].

In [13], multipath information ia used together with the image theory to locate either an emitter or a target, showing the feasibility to perform location using only one sensor. In electromagnetic localization systems the performance is disturbed by the multipath rays, where the non-line-of-sight rays affect the time measurements.

The emitter localization can also be performed using only one sensor, as explained in [14], where there is an algorithm that estimates the time-of-arrival (TOA) and makes a so-called “wall association” for assigning the reflected ray with some defined wall to deal with sensing and through-the-wall (TTW) radar inside buildings. Another passive location approach, described by O’connor et al. [15], also uses multipath characteristics of the scenario to locate the emitter, using the time of arrival of the NLOS rays, introducing a clustering procedure to match the real measurements with the simulated one.

Doğançay [16] introduced a new pseudo-linear estimator for 3D Target Motion Analysis (TMA). The authors presented the 3D pseudo-linear estimator, which is derived as a small noise approximation to the maximum likelihood estimator and contains the 2D pseudo-linear estimator for the XY-coordinates of the target motion parameters, and a Z-coordinate least squares estimator is proposed. In addition to that particular angle, measurement is invoked to implement the weighted instrumental variable and also maintain a strong correlation between the matrices in the case of considerable measurement noise. The target localization problem is proposed by the difference between the 2D and 3D angle localization using a pseudo-linear location estimator. MLE does not have the closed form solution and is computationally more intensive and complicated when used for large-scale monitoring.

Several algorithms were tested by Qi et al. [17] to identify the NLOS to improve the localization. First, an algorithm performs a test ratio based on the cumulative distribution of the received signal envelopes with a predefined bound, and other approaches observe the level crossing rate and the average fade duration. The Cramer–Rao bound (CRB) has been analyzed for several geolocation schemes in the presence of the NLOS, showing that the Fisher Information Matrix (FIM) of a hybrid scheme using the RSS and the TDOA can be acquired by the superposition of the FIM from both schemes.

A new positioning novel framework presented in [18] showed how to estimate the passive source localization accurately using joint sensor measurements that are AOA and TDOA, with different independent sensors being collocated at the reference positions of arrays. The bearing and time delay estimation is considered, and the signal gain information can also be used when the acoustic sensor array networks measure source signals. This paper explores the potential performance improvement of localization using joint sensor measurements, but the problem is relatively low localization accuracy due to the high estimation of measurement error.

An optimal TDOA sensor-pair placement is discussed in [19] where two categories of sensor pairing methods are illustrated, namely centralized and decentralized, and the optimal sensor geometries are derived for both stationary and movable cases. In the case of moving sensors, the geometries plan is investigated using Extended Kalman Filter (EKF). Additionally, communication constraints and sensor motion are extended for optimal sensor path planning problem. This paper only studies the single step trajectory optimization. This case is not suitable for all areas (e.g., large- and small-scale area monitoring).

A new two-step algorithm was proposed by He et al. [20]. In the first step, the positioning algorithm combines Taylor Series method Semidefinite Programming (SDP) to achieve the global convergence and estimation accuracy. In the second step, a constrained least squares method provides the benefit of low complexity and fast convergence for maintaining the system performance. Besides, a new receiver selection method is invoked to reselect the sensor for path planning and to improve the estimation accuracy.

A localization approach was presented by Tran et al. [21], where a relaxation-based technique using the algorithms local neighborhood multilateration and convex optimization. The network scenario is divided into overlapping regions, improving localization accuracy by using a set of beacons and extrapolating unknown node locations from the beacon locations. In [22], an extension of this approach using statistical learning theory is applied to the location, based on the Support Vector Machine (SVM).

In all the mentioned studies, it is common to use the Euclidean geometrical properties to perform location finding, using the topology implicit in sets of sensor readings and locations can be exploited to using signal-based function spaces that are useful for the prediction of unknown emitter locations.

### 1.2. Research Contributions

The main contributions of our research work are to improve the TDOA location system in NLOS situation using channel impulse estimation in time-domain and ray tracing simulations, building a multipath exploitation framework that tries to include suite-specific reflection as an extra layer of information.

Much of the previous literature deals with the NLOS as outliers in the location algorithm. To overcome this problem and to enhance the system efficiency, we propose a novel algorithm that uses long-term measurements to extract the information about reflection points in the scenario and build a classification framework using the machine learning approach. In our proposed work, we first calculate the time differences several times using an improved cross-correlation approach and a WLS algorithm to estimate the emitter position. The proposed method was applied to test the improvement in localization precision performance in NLOS situation.

This paper is structured as follows. Section 2 discusses the problem formulation, including the signal data model and the Channel Impulse Response estimation. An experimental setup, showing the application of the proposed localization method and including the results of the simulation setup is discussed in Section 3. Finally, Section 4 summarizes the principal aspects of this paper and presents possible research direction using the proposed methodology.

## 2. Problem Formulation

This section presents the data-model for localization of an RF source using a set of sensors in an outdoor scenario, where the TDOA systems have the advantage of AoA because they can use a single antenna per sensor. On the other hand, they need to use a high capacity data-link to connect the stations to keep the synchronization reference among them; thus, the TDOA data model can be described in a unified framework based on the derivation of [3]. In our case, we assume that there is a clock synchronization in all the observers deployed.

### 2.1. Data Model for TDOA Localization

Assuming that each sensor records the signal, a sample is sent for the “reference station”, which performs signal processing with the signal received; the time differences are the lags in the peak in the cross-correlation function of the complex base-band (CBB) signal received. It is possible to use the term “range-difference” of arrival (RDOA) instead of “time-difference” between the emitter and the sensors, by multiplying the TDOA by the electromagnetic propagation speed.

From the geometric interpretation, a hyperbola is a set of points, such that the absolute range difference between the two fixed points, known as foci, is constant. If we assume the points with a constant range difference between two fixed observers as hyperbola in the 2-D space, then the location of the target lies in the intersection of the lines.

The scenario is characterized by an emitter at the point $x_{e},y_{e},z_{e}$ and *l* sensors deployed at the points $x_{l},y_{l},z_{l}$; the target sends a signal at the unknown time $t_{0}$, and the *l*th sensor receives it at time $t_{l},l=1,2,\ldots ,L$ with L≥3. There are L(L−1)/2 distinct TDOAs from all possible sensor pairs, denoted by: $t_{k,l}=\left(t_{k}-t_{o}\right)=t_{k}-t_{l},k,l=1,2,\ldots ,L$, with k>l. However, there are only (L−1) non-redundant TDOAs.

**Figure 1:**
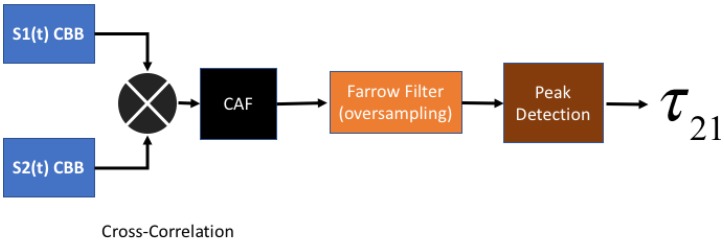
TDOA Estimation based on the Complex Baseband (CBB) signals arriving at Sensors 1 and 2.

**Figure 2:**
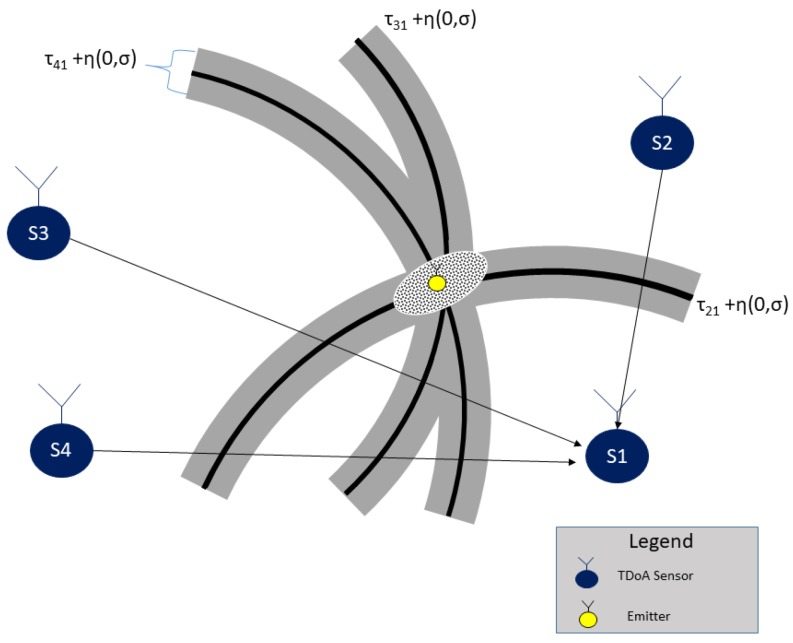
Location error caused by multipath situation in TDOA measurements.

Assuming only the (L−1) non-redundant TDOAs for source localization [23] and considering the first sensor as reference, they are $t_{i,1}$,l=2,3,…,L. The range difference can be deduced using the TDOAs as:

(1) $$r_{TDOA,l}=d_{l,1}+n_{TDOA,l},l=2,3,\ldots ,L$$

where

(2) $$d_{l,1}=d_{l}-d_{1}$$

and $n_{TDOA,l}$ is the range difference error in $R_{tdoa,l},$ which is proportional to the disturbance in $t_{l,1}$. Figure 1 shows the steps to extract a time-difference from the complex baseband signal received by two sensors. The small error in the time difference measurements affects the position estimation precision, as can be seen in Figure 2. The noise in range measurements affects the location performance.

Using TDOA model in matrix form, we have:

(3) $$r_{TDOA}=f_{TDOA}\left(x\right)+n_{TDOA},$$

where

(4) $$r_{TDOA}={\left[r_{TDOA,2}r_{TDOA,3}....r_{TDOA,L}\right]}^{T}$$

(5) $$n_{TDOA}={\left[n_{TDOA,2}n_{TDOA,3}....n_{TDOA,L}\right]}^{T}$$

and

(6) $$f_{TDOA}\left(x\right)=d_{l}=\left[\begin{array}{c} \sqrt{{\left(x-x_{2}\right)}^{2}+{\left(y-y_{2}\right)}^{2}-{\left(z-z_{2}\right)}^{2}}-\sqrt{{\left(x-x_{1}\right)}^{2}-{\left(y-y_{1}\right)}^{2}-{\left(z-z_{1}\right)}^{2}}\\ \sqrt{{\left(x-x_{3}\right)}^{2}+{\left(y-y_{3}\right)}^{2}-{\left(z-z_{3}\right)}^{2}}-\sqrt{{\left(x-x_{1}\right)}^{2}+{\left(y-y_{1}\right)}^{2}-{\left(z-z_{1}\right)}^{2}}\\ .\\ .\\ .\\ \sqrt{{\left(x-x_{L}\right)}^{2}+{\left(y-y_{L}\right)}^{2}-{\left(z-z_{L}\right)}^{2}}-\sqrt{{\left(x-x_{1}\right)}^{2}+{\left(y-y_{1}\right)}^{2}-{\left(z-z_{1}\right)}^{2}} \end{array}\right]$$

The estimation of source localization position using TDOA measurements is therefore to find **x** given the $r_{TDOA}$, assuming that $n_{TDOA}$ is zero-mean and Gaussian noise in the range measurements with Probability Density Function (PDF), for $r_{TDOA}$, denoted by $p\{r_{TDOA}\}$ with the following structure:

(7) $$p\left(r_{TDOA}\right)=\frac{1}{{\left(2\pi \right)}^{(L-1)/2}{\left|C_{TDOA}\right|}^{1/2}}e^{\left(-\frac{1}{2}{\left(r_{TDOA}-d\right)}^{T}C_{TDOA}^{-1}\left(r_{TDOA}-d\right)\right)},$$

where $C_{TDOA}$ is the covariance matrix for $r_{TDOA}$.

It is also possible to use $r_{TDOA}∼N\left(d_{1},C_{TDOA}\right).$ Since all TDOAs are determined with respect to the first sensor, $n_{TDOA},l=2,3,\ldots ,L$ are correlated. As a result, $C_{TDOA}$ is not a diagonal matrix [23]. When the time difference of arrival (denoted here by τ) measured at two or more widely dispersed sensors are used to locate emitters, the methodology presented by Poisel [24] yields the position estimation in closed form using TDOA measurements. The TDOA signal models and their basic positioning principles can be generalized as:

(8) r=f(x)+n,

where *r* is the measurement vector, *x* is the source position to be determined, f(x) is a known nonlinear function in *x*, and *n* is an additive zero-mean noise vector.

### 2.2. Source Localization Algorithms

The algorithms for source localization can use a nonlinear or a linear approach. In general, the nonlinear methodology [25,26] solves Equation (1) to find *x* by minimizing a cost function based on the error estimation with least squares (LS) or the weighted least squares (WLS) formulation:

(9) $$e_{nonlinear}=r-f\left(\tilde{x}\right).$$

$\tilde{x}={\left[\tilde{x}\tilde{y}\right]}^{T}$ is the optimization variable for *x*, which corresponds to the ML estimator. As described in [3], it is possible to convert Equation (9) into a set of linear equation in *x*, as:

(10) b=Ax+q,

where *b* and *A* are available and *q* is the noise vector. Then, the error is:

(11) $$e_{nonlinear}=b-A\tilde{x}$$

Applying the LS or WLS techniques on Equation (11) results in the LLS [27,28], WLLS [29,30] and subspace [31,32] estimators.

In the linear approach, the basic idea is to “linearize” the nonlinear expression of position estimation into a set of linear equations, assuming that the disturbances caused by the measurement errors are small and with zero-mean. The linearization can use three approaches: LLS, WLLS, and subspace estimators. The LLS approach attempts to reorganize Equation (11) into linear equations in *x*; the position is then obtained using the conventional LS technique. The estimation is done using the TDOA structure from Equations (5) and (6) as:

(12) $$r_{TDOA,l}+\sqrt{{\left(x-x_{1}\right)}^{2}+{\left(y-y_{1}\right)}^{2}}=\sqrt{{\left(x-x_{1}\right)}^{2}+{\left(y-y_{1}\right)}^{2}}n_{TDOA,l}^{2},l=2,3,\ldots ,L.$$

(13) $$\begin{array}{cc} r_{TDOA,l} & +\sqrt{{\left(x-x_{1}\right)}^{2}+{\left(y-y_{1}\right)}^{2}+{\left(z-z_{1}\right)}^{2}}\\ & =\sqrt{{\left(x-x_{1}\right)}^{2}+{\left(y-y_{1}\right)}^{2}+{\left(z-z_{1}\right)}^{2}}\\ & +n_{TDOA,l}^{2}\;with\;l=2,3,\ldots ,L. \end{array}$$

The noise is modeled as the modified component:

(14) $$m_{TDOA,l}=n_{TDOA,l}^{2}+2n_{TDOA,l}\sqrt{{\left(x-x_{1}\right)}^{2}+{\left(y-y_{1}\right)}^{2}+{\left(z-z_{1}\right)}^{2}}.$$

In short, the matrix structure for TDOA localization is:

(15) $$A=2\left[\begin{array}{cccc} x_{1}-x_{2} & y_{1}-y_{2} & z_{1}-z_{2} & -r_{TDOA,2}\\ x_{1}-x_{3} & y_{1}-y_{3} & z_{1}-z_{3} & -r_{TDOA,3}\\ \vdots & \vdots & \vdots & \vdots \\ x_{1}-x_{L} & y_{1}-y_{L} & z_{1}-z_{L} & -r_{TDOA,L} \end{array}\right]$$

(16) $$\theta ={\left[\begin{array}{cccc} x-x_{1} & y-y_{1} & z-z_{1} & R_{1}, \end{array}\right]}^{T}$$

(17) $$q={\left[m_{TDOA,2},m_{TDOA,3},\ldots ,m_{TDOA,L}\right]}^{T},$$

and

(18) $$b=\left[\begin{array}{cccc} r_{TDOA,2} & -{\left(x_{1}-x_{2}\right)}^{2} & -{\left(y_{1}-y_{2}\right)}^{2} & -{\left(z_{1}-z_{2}\right)}^{2}\\ r_{TDOA,3} & -{\left(x_{1}-x_{3}\right)}^{2} & -{\left(y_{1}-y_{3}\right)}^{2} & -{\left(z_{1}-z_{3}\right)}^{2}\\ \vdots & \vdots & \vdots & \vdots \\ r_{TDOA,L} & -{\left(x_{1}-x_{L}\right)}^{2} & -{\left(y_{1}-y_{L}\right)}^{2} & -{\left(z_{1}-z_{L}\right)}^{2} \end{array}\right].$$

The data model for estimating a position of a radio-frequency emitter using TDOA with either nonlinear or linear approach provides good results if the errors are small and the noise can be modeled as a Gaussian distribution with zero-mean. The literature [24] shows that the estimation performance of the ML and constrained WLLS methods can achieve the CRLB.

### 2.3. Effect of NLOS in TDOA Estimation

**Figure 3:**
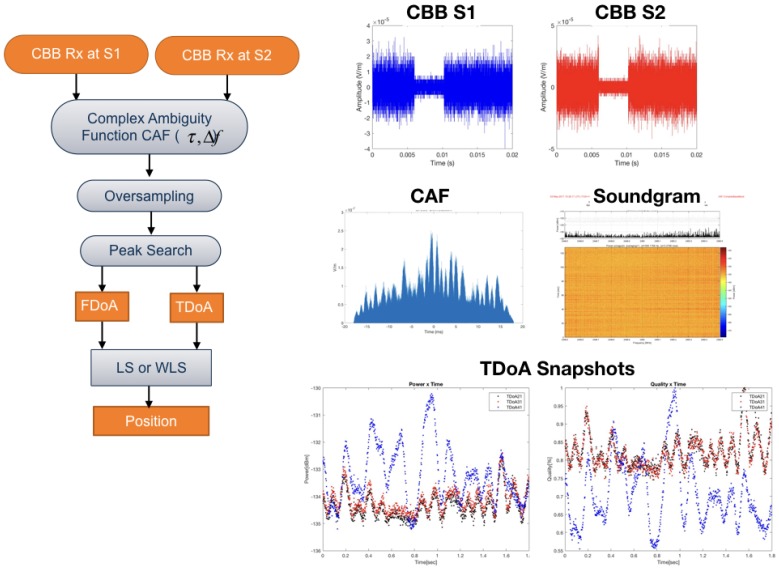
TDOA processing in Sensor 1 and 2.

The computation of the time difference between the signals received by the sensors is schematically shown in Figure 3. The complex baseband signal recorded at Sensor 1 and 2 are cross-correlated and oversampled using Farrow filters, as described by Kolb et al. [33], to increase the spatial resolution. Finally, a time delay is obtained with the “lag” index of the peak in Complex Ambiguity Function (CAF).

The generalized cross-correlation function in phase (GCC-PHAT) is performed and a peak detection operation is applied to find the maximum and give $\tau_{21},\tau_{31},\tau_{41}$. With the three time differences extracted by the cross-correlation operation, either the linear or nonlinear method is performed to estimate the position.

The point now is to model the effect of multipath in the estimator. One possibility is to include the multipath in the signal data model and the CRB, as presented in [34], as an “extra error” in the estimation. As a nonlinear operation, the imprecision caused by the multipath will give some “bias” in the position estimation, but, in a real scenario, the multipath effect is a highly non-linear operation, where the channel impulse response will be time-variant with rays showing up and disappearing during the time-frame of the measurements. Therefore, the NLOS error modeled here is considered a “small” random variation in time differences. This approach is feasible only when the specular components (SC) are well defined and more significant than the Dense Multipath Components (DMC) [35]. For this reason, the framework developed in this paper is suitable for suburban and campus-like outdoor scenarios, where the buildings are spaced and low, typically with five floors.

(19) $$e_{nonlinear}=e_{noise}+e_{NLOS},$$

**Figure 4:**
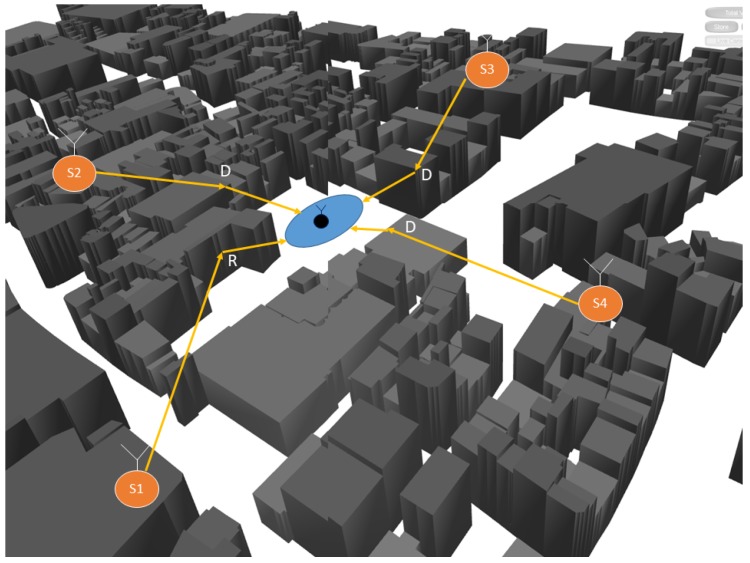
Error caused by NLOS situation in TDOA systems.

**Figure 5:**
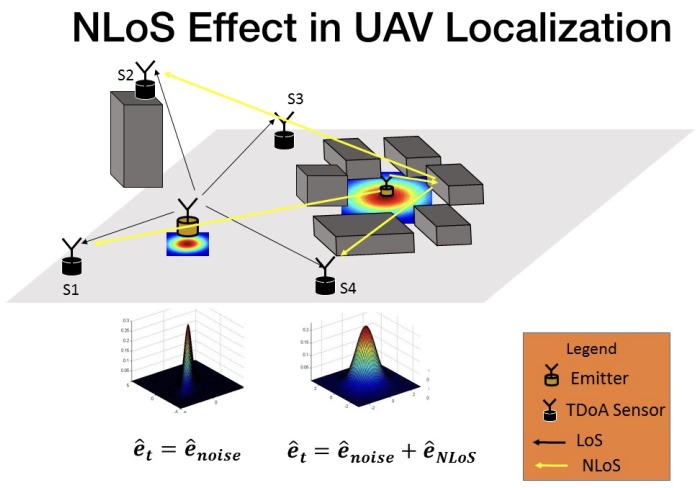
The error produced in TDOA location by multipath.

This section has formulated the data model to support the problem of estimating a radio-frequency emitter in an NLOS scenario. The TDOA discussed in this paper is, therefore, a standard system but with extra noise in the estimation performance that leads to a wrong position. As can be seen in Figure 4, the TDOA system performance is affected by the buildings, affecting the position estimation, the situation can also schematic presented in Figure 5, where the multipath presents an additional error in measurements.

## 3. Proposed Method

This section describes the method proposed to enhance a TDOA localization system when the emitter is in NLOS position, which is a typical situation of the outdoor channel characteristics and cannot be avoided. Some methods deal with multipath assuming that they are outliers, but this approach is not feasible in a typical suburban scenario.

**Figure 6:**
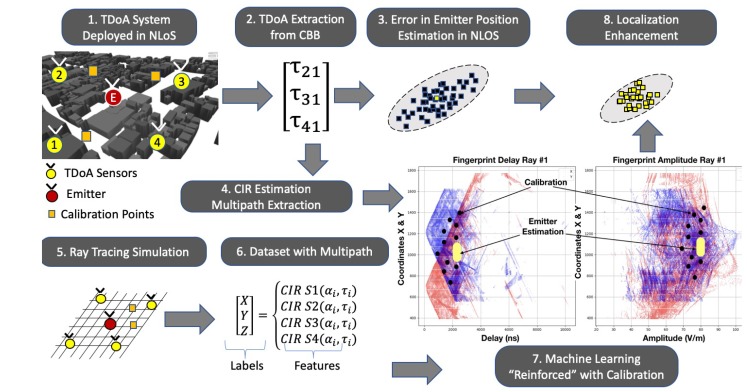
Main steps of the proposed method.

The main blocks in the method proposed are presented in Figure 6 and are further discussed in this section. First, a TDOA system is deployed in an NLOS scenario, and the multipath effects corrupt the time-differences extracted from the complex baseband signals that arrived in each sensor. In parallel, with the sensor position information and the scenario obstacles, several ray-tracing simulations were performed to extract a spatial fingerprint of the channel impulse response in each point. The output of the ray-tracing simulation feeds a dataset of that defines a multipath fingerprint where the coordinates act as “abels”, and the rays multipath information acts as “features”. A machine learning framework is performed using the calibration points multipath information to “reinforce” the machine learning engine. Finally, the TDOA localization position estimation is enhanced with the proposed framework.

The idea is to bind the ray-tracing simulation and machine learning to perform a multipath pattern matching between the simulated channel impulse components and the signal recorded by the sensors to enhance the performance of the localization system in a typical suburban scenario.

The inputs for the localization problem are the sensor position, which is usually known; the signal received; and the scenario description. Using this information, we tried to improve the performance by adding a multipath fingerprint of the NLOS patterns. As described in Section 2, when the scenario is suburban or campus-like, the SC are usually stronger than the DMC. In this case, the NLOS components give a small “bias” in the position estimation of the TDOA System, adding an extra error in the time differences. It might be possible, under these circumstances, to make a hybrid approach using ray-tracing simulations, channel impulse response estimation, and machine learning to enhance the TDOA localization performance.

**Figure 7:**
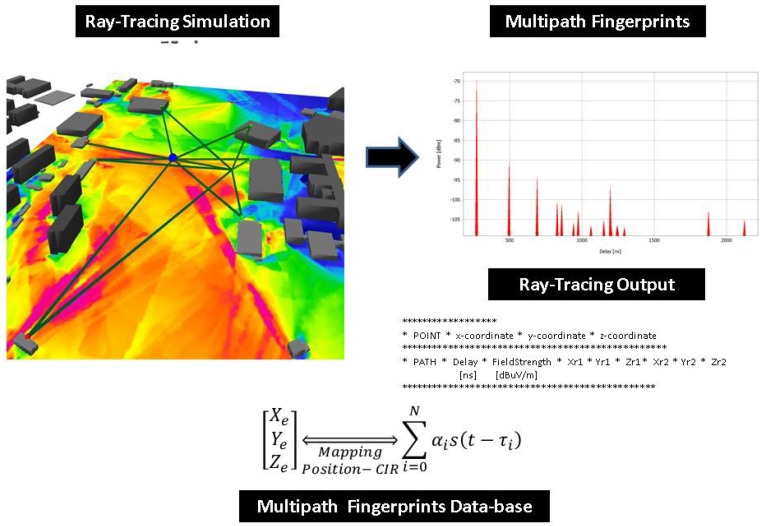
Multipath fingerprint database using ray-tracing simulation.

**Figure 8:**
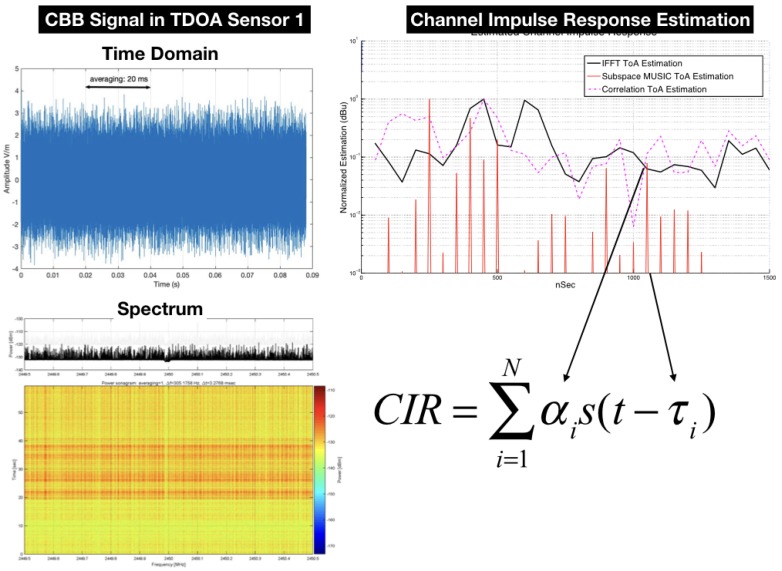
Channel impulse estimation in each TDOA sensor.

The method is schematic, as presented in Figure 7 and Figure 8. First, the ray tracing performs several simulations of the outdoor scenario, and the output of the simulation is a description of the paths from each component of the signal that connects the emitter position to the receiver point. A multipath fingerprint database is associated with each point of the simulation domain to a specific channel impulse response; this procedure is done for each of the deployed TDOA sensors. The second step is to estimate the CIR to extract the multipath information ($\alpha_{1},\tau_{i}$) from the receiver signal in each sensor position. Finally, a neural-network based on a machine-learning engine performs the estimation of the emitter position, enhancing the TDOA performance in the outdoor NLOS situations. Depending on the precision of the obstacles description and the material properties included in the simulation, the ray tracing gives a reasonable representation of the specular components that characterize the transmitter–receiver relationship.

For each possible point for the emitter in the simulation, the amplitude and delays for the multipath components in each TDOA sensor are given by:

(20) $$\begin{array}{cc} S_{1}=\sum_{i=1}^{M}\alpha_{i}x\left(t-t_{i}\right), & S_{2}=\sum_{i=1}^{M}\alpha_{i}x\left(t-t_{i}\right)\\ S_{3}=\sum_{i=1}^{M}\alpha_{i}x\left(t-t_{i}\right), & S_{4}=\sum_{i=1}^{M}\alpha_{i}x\left(t-t_{i}\right). \end{array}$$

This multipath fingerprint dataset makes the association of each point of the simulation domain to a specific multipath pattern related to each deployed TDOA sensor. The next step is to take the signal received by each sensor and make the Channel Impulse Response (CIR), where the information ($\alpha_{1},\tau_{i}$) extracted from the Complex Baseband (CBB) received signal is associated with a defined position in the scenario.

The matching procedure between the estimated CIR, and the multipath dataset is performed using a neural-network based on a machine-learning engine. The estimation of the position of the emitter is used to add a new layer in the localization performance of the deployed system, enhancing the TDOA performance in the outdoor scenario even in NLOS situation.

The multipath dataset is performed using a neural-network-based with a machine learning engine. The emitter position estimation is used to add a new layer in the localization performance of the deployed system, enhancing the TDOA performance in the outdoor scenario even in NLOS situation.

The matching procedure and the specific details for the ray-tracing simulation, the channel impulse estimation and the machine learning in the method are explained in the following sections.

### 3.1. Multipath Dataset Using Ray Tracing

Ray tracing is an electromagnetic simulation tool for modeling signal propagation. Assuming the geometric optics approximation, we can trace the ray propagation paths in a defined scenario, following Degli-Esposti [36]. The term “ray tracing” fingerprint is usually associated with the “radio maps” of Received Signal Strength (RSS) in a coverage prediction. However, this approach needs to take into account the output power of the emitter to perform the position estimation [37].

The RT simulation provides the "site-specific” channel impulse response; thus, as soon as the position of each sensor is defined, it is possible to obtain the multipath information of each point of the scenario. The specular component paths, including all reflection and diffraction points, are available at the end of the ray-tracing simulation. Depending on the desired number of ray interactions, image theory allows identifying the reflection points and the virtual nodes, thus all possible rays in the simulation domain can be estimated.

The spatial characterization of the path-loss is based on the premise that there is a one-to-one relationship between the signals received at the base stations and the emitter location [3]. The fingerprint can be extracted from the received signals and serves as a unique identifier of the emitter location that is reduced to a pattern recognition problem, where the location is determined by matching the extracted fingerprint to the fingerprint database.

The Figure 8 shows the multipath fingerprints produced by ray trace simulation from Sensor Position 1. As shown in the figure, the output of the simulation describes each path from each point in a grid that is defined in the scenario, not only the amplitude and the delay ($\alpha_{i},\tau_{i}$) but also the reflection points and the angular information of each ray. The ray tracing output also gives information about the multipath components propagation mechanisms, reflection, diffraction or scattering.

**Figure 9:**
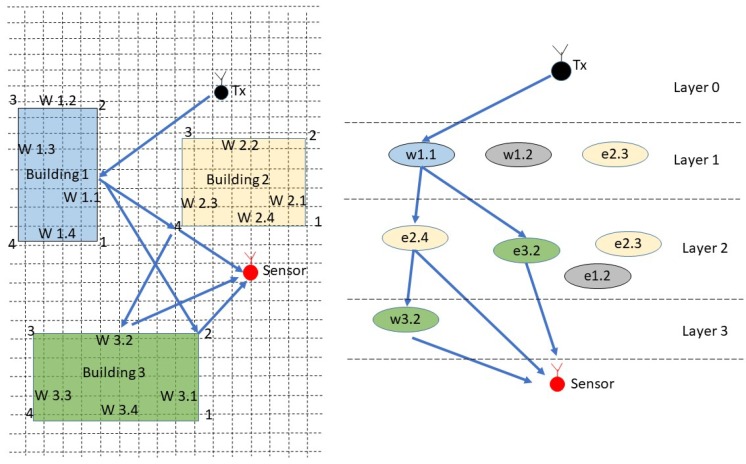
Schematic representation of walls and edges in ray-tracing simulation.

Following the approach of Fuschini et al. [38], the ray-tracing scenario can be decomposed into walls, edges, and the “view tree”, which represent the emitter–sensor interactions, and are the basis for the visibility matrix where each interaction is considered as a layer in a multilayer scheme. Therefore, the RT gives the information about each ray path, describing the edges and walls touched by the rays in the emitter-sensor path. The Figure 9 shows how the three simulation structure is related with the obstacles walls and edges, the rays path are defined by interaction layers, given a complete description of the NLOS components that connect the emitter with the sensor position.

With the output file of ray tracing, it is possible to search for the given positions where the reflectors bounce off the rays before arriving at the sensors. This works similarly to extracting the “field of vision” in an optics ray.

The ray tracing can be combined with the multipath fingerprints from the scenario by using ray-tracing simulation because the effects of the buildings and obstacles are also included in the simulation. However, the performance is highly dependable on the details given in the scenario setup. In practical outdoor implementations, the buildings are represented by simpler structures, where window and door details are extracted out from the simulation. For a suburban outdoor scenario with simple buildings, the ray trace gives reasonable information about the main specular components in the propagation channel.

The NLOS situation is an inherent part of the channel characteristics and cannot be avoided; therefore, the methods that extract the NLOS TDOA measurements by assuming NLOS are outliers are not feasible in a typical suburban scenario. The localization problem starts with the sensor position, which is usually known; the signal received; and the scenario description or characterization. With this information, we improve the TDOA performance by adding a multipath fingerprint using the NLOS patterns.

### 3.2. Channel Impulse Response Estimation

In signal processing theory, there are several well-established methods to recover the multipath components [39], namely, the amplitude and the time delay, from the received signal. There are two approaches to CIR extraction depending on whether the sensors know or do not know the type of the emitted signal: blind estimation and the estimation of a known signal.

The practical TDOA localization systems for outdoor scenario use an up-sampling procedure with “farrow filters” to improve the range resolution Kolb et al. [33] but the traditional Generalized Cross-Correlation algorithm (GCC) has lower resolution in time and cannot give the TDOA of closest delayed multipath signals.

Assuming that the emitted signal is known, it is necessary to estimate at least the five most robust specular components, extracted from the received signal; the resolution achieved in the CIR estimation is directly dependent of the signal bandwidth and the sampling frequency used. The CIR estimation techniques can be grouped into correlation-based methods, deconvolution methods, maximum likelihood (ML)-based methods, subspace-based methods, and blind signal separation methods.

The multipath channel can be modeled as:

(21) $$h\left(t\right)=\sum_{m=1}^{M}\alpha_{m}\delta \left(t-\tau_{m}\right).$$

where *M* represents the number of paths, and $\alpha_{m}$ and $\tau_{m}$ are the complex attenuation and propagation delays of the *m*th path, respectively. It is also assumed that $\tau_{1}<\tau_{2}<\ldots <\tau_{m}<$, thus $\tau_{1}$ represents the delay of the first received ray, as follows:

(22) $$x\left(t\right)=\sum_{m=1}^{M}\alpha_{m}s\left(t-\tau_{m}\right)+\nu \left(t\right).$$

Taking s(t) as the transmitted signal, with bandwidth of *B*, center frequency of $f_{c}$, and duration of $T_{0}$, ν(t) is assumed to be white Gaussian noise with zero-mean uncorrelated with s(t). It is also possible to use the deconvolution to estimate the CIR, based on “inverse filters”, where the receiver signal x(t) can be represented by a convolution between the transmitted signal and the CIR:

(23) x(t)=s(t)⊗h(t)+ν(t).

Alternatively, in the frequency domain:

(24) X(f)=S(f)H(f)+V(f).

The CIR can be estimated using:

(25) $$h\left(t\right)=ifft\left\{X(f)/S(f)\right\}.$$

This operation can also be done in the frequency domain, using the signal model in vector format:

(26) X=SH+V.

To estimate the CIR using this approach is the process to recover the H, which reproduces the multipath rays composition characteristic of the channel because each of the non-zero elements in the matrix is the amplitude of the ray and a defined time delay position. Applying the covariance matrix of Y, we have:

(27) $$R_{y}=EYY^{H}=UR_{A}U^{H}H+\sigma_{\nu }^{2}I.$$

where $R_{A}=EAA^{H}$. We can extract the parameters, $\left(\alpha_{i},\tau_{i}\right)$, that define each ray using MUSIC (MUltiple SIgnal Classification) signal processing to find the biggest eigenvalues of $R_{Y}$.

Based on the orthogonality between signal and noise subspace, this approach can handle closed spaced multipath components. This method is used in the localization procedure to extract the channel parameters to make it possible to localize the emitter. Splitting the signal into the signal subspace and the noise subspace, following the approach of Fleury et al. [40], the pseudo-spectrum using MUSIC is:

(28) $$S_{MUSIC}=\frac{1}{{\left|Pu\left(\tau \right)\right|}^{2}}.$$

**Figure 10:**
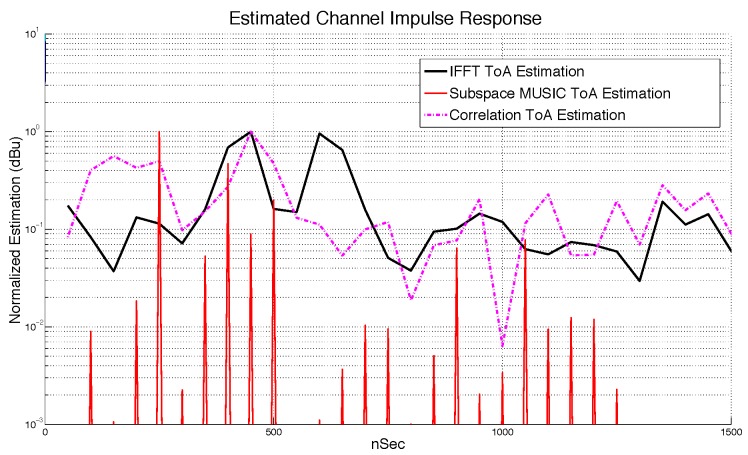
Performance in estimation of ($\alpha_{i},\tau_{i}$) for CIR.

The three approaches introduced here can be seen in Figure 10, where subspace technique gave the best result, regarding the ability to discriminate closed spaced multipath rays.

Zhu et al. [41] showed that further resolution could be achieved using the sparse recovery framework and union spaces. In this study, we dealt with an outdoor suburban scenario, where the MUSIC in time-domain was used to estimate the five most robust specular components from the CIR in each sensor.

For the suburban scenario considered in this study, the five specular components were estimated using the MUSIC in time-domain.

### 3.3. Localization Using NLOS Ray-Tracing Fingerprints

**Figure 11:**
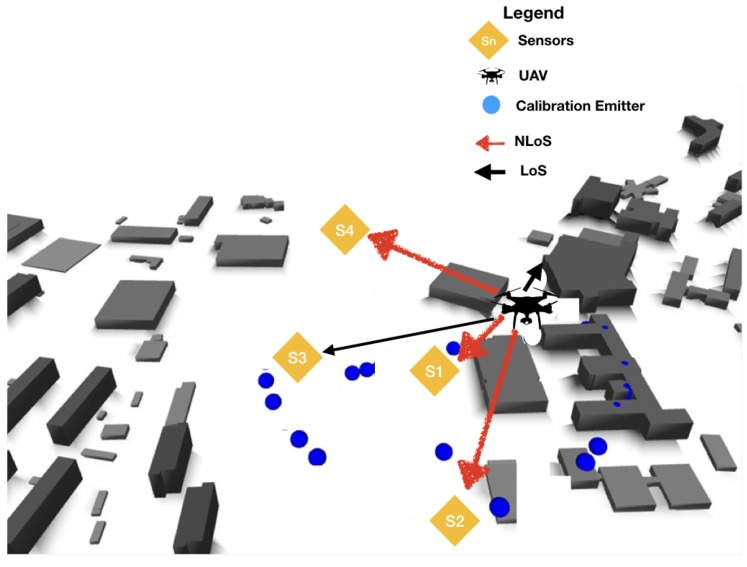
TDOA location scenario in NLOS.

In this scenario, besides the TDOA sensors, we also have the reference ($N_{r}$) and anchors points $N_{t}$ to help the estimation of the emitter position, which are labeled as an “unknown point” $N_{u}$. The known positions from anchors nodes are $T_{i}$ with $i\in 1,\ldots ,N_{t}$, and from the reference nodes are $R_{j}$ with $j\in 1\ldots ,N_{r}$. Finally, the emitter positions are the unknown nodes that are estimated and defined by $U_{k}$ with $k\in 1,\ldots ,N_{u}$, as shown in Figure 11. Performing the CIR estimation from the reference points gives a set of calibration values to correct the ray-tracing dataset.

A fingerprinting approach normally takes a off-line calculation where the multipath information is collected by using the reference node signal, $r_{j}$, at the *j*th reference node position, $R_{j}$, for every $j\in 1,\ldots ,N_{r}$. With this information, the next step is to find a model that can better describe the relationship among the reference node position, the ray-tracing simulations, and the estimate CIR. This operation acts as a refinement in the dataset obtained by ray tracing and is considered in the machine-learning framework as a “reinforcement” learning procedure.

In a vector format, the model maps the CIR and ray-tracing fingerprints, as $R_{j}\approx f\left(r_{j}\right)$; the model uses as many measurements and reference point as needed to achieve a certain precision level, until a there is a feasible model in vector format f(.)

### 3.4. Machine Learning in Multipath Fingerprint

As described by Alsheikh et al. [42], machine learning is an interdisciplinary field of applied mathematics, which relies on developing a hypothesis of creating the model (as opposed to an algorithm in computer science or methods or formula in mathematics) and tries to improve it by fitting more data into the model over time. The idea is to use the multipath information to predict geolocation of the emitter; regression is one of the choices for prediction, because it is a class of supervised algorithms [43] that attempts to establish a continuous relationship between a set of dependent variables (geolocation coordinates) and set of other independent variables (multipath fingerprints, $\alpha_{i},\tau_{i}$).

**Figure 12:**
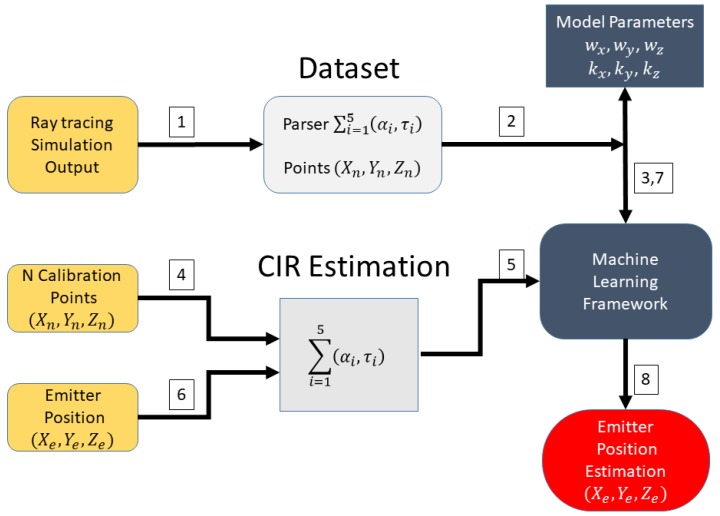
Machine learning ray-tracing dataset refinement.

**Figure 13:**
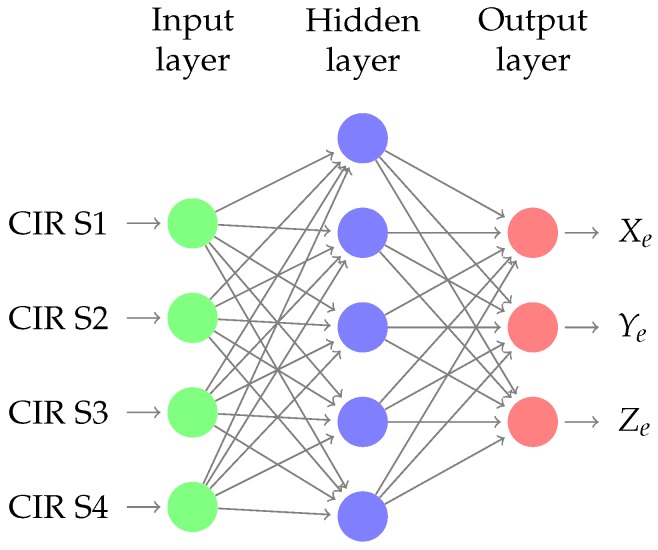
Neural network for position estimation.

**Figure 14:**
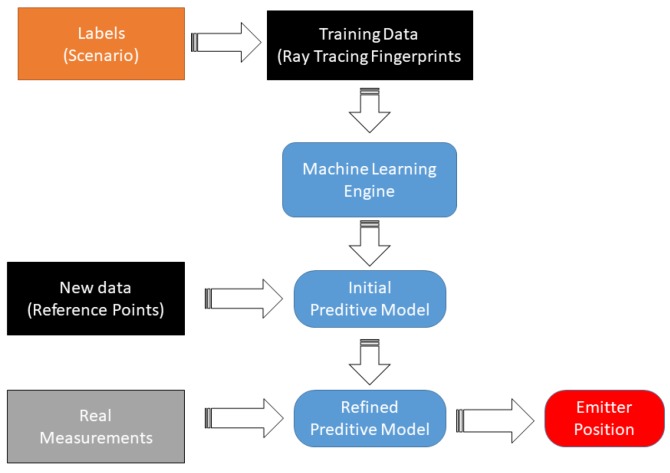
Machine learning framework based on RT simulation.

The dataset produced by ray tracing and the refinement procedure is schematically shown in Figure 12. First, the ray tracing feeds a dataset that is “parsed” to label each point to a specific CIR; then, a model parameter is extracted from the dataset to generalize the multipath patterns classification. To refine the dataset produced by ray tracing, a channel impulse estimation using MUSIC in time-domain is performed to extract the five strongest specular components from the CBB signal collected from a set of calibration points. The Figure 13 show the schema of the Neural Network used, where the input layer are the CIR estimation of each sensor and the emitter position coordinates are the output. This multipath “fingerprints” are used to adjust the model parameters. Finally, a CIR estimation is done using the signal from the emitter, and the position is estimated. The Figure 14 shows the machine learning reinforcement procedure based on RT simulation and multi path fingerprints.

There are some central aspects of using ray tracing and real measurements encapsulated in the machine-learning engine. First, we tried to use learning classification to approximate the position estimation as a target function for optimization; (semi-)supervised machine learning makes an approximation to find a target function (*f*). This function should be able to map input variables (*X*) that are the multipath components ($\alpha_{i},\tau_{i}$) to an output variable (*Y*) that is the emitter position.

(29) $$Y=f\left(X\right)=f(X\left(\alpha_{i},\tau_{i}\right)).$$

Another consideration in machine learning is to target function from the training data to check how well the model generalizes to new data input. That is why generalization is necessary: the collected data work as a sample, and are incomplete and noisy.

The idea is to make a fingerprint that improves continuously with an anchor emitter that performs a recursive averaging and recalculation of the multipath fingerprint patterns. The hypothesis is developed based on the knowledge of the relationship between dependent and independent variables in TDOA including NLOS identification. We used a set of tools as a machine learning framework to develop and test the application:

- Jupyter Notebook version 5.1.0 (http://jupyter.org);
- Python version 3.6.3 (https://www.python.org);
- Python numpy version 1.13.3 (http://www.numpy.org);
- Tensorflow version 1.4.0 (http://tensorflow.org); and
- CentOS version 7.4.1708 (https://www.centos.org) in Google cloud (http://cloud.google.com).

At this point, the approximation, not only in the scenario description but also in the in multipath information from ray tracing can play an essential role in the machine-learning framework. Thus, the ray description should be good enough to establish the model, but cannot be so precise as to lose the generalization features, which could happen because we try to describe the learning of the target function from training data as inductive learning.

These effects are well-known in the literature as over- and under-fitting of the dataset, and are the two biggest causes for poor performance of machine-learning algorithms [44]. The task of a good machine-learning model is to generalize well from the training data to any data from the problem domain. The multipath based on ray-tracing dataset should present some typical channel characteristic. If the multipath description is good enough, the supervised machine learning algorithms can try to find the best approximation to the unknown underlying mapping function for the output variables given the input variables.

The machine learning and kernel methods can work as supervised, unsupervised or semi-supervised learning frameworks. In our approach, the ray tracing gives the initial dataset for the ML and the CIR estimation of the reference or anchor nodes multipath works as a “reinforcement” or supervised learning. The hybrid approach uses real measurements and simulations to build the dataset, which can be classified as semi-supervised learning.

The problem tackled was predicting the emitter position of the point, i.e., its X, Y and Z coordinates, using linear regression when the delay and amplitude of the five multipath rays were given. The network had ten inputs, i.e., five rays. Neural networks, linear regression, and random forest algorithm were used to achieve the result, and the random forest proved to be the best one.

One of the problems in the proposed model was the points that have less than five paths. It was necessary to assign them with 0 s to given same length input to the network, thus biases were huge and weights were low, which caused such an imbalance that the neural network’s mean squared error loss was high. Then, a set of hyper-parameters was used to tune the linear regression [45], giving better results.

The method implemented uses the ray-tracing fingerprints to enhance the position estimation performance continuously, using known anchor emitters and the multipath fingerprint patterns. The hypothesis is developed based on the knowledge of the relationship between dependent and independent variables in TDOA including NLOS identification. In our formulation, we use the five strongest rays that arrive at each point of the simulation domain. The proposed algorithm is summarized in Table 1.

In short, the proposed method combines CIR estimation, ray-tracing simulation, and machine learning to match the amplitude and delay information to a defined position. If one only uses the amplitude or the received power information, also available in ray tracing, the method is similar to RSSI [3]. The fingerprints produced by five rays in four different receivers add more dimensions in the matching procedure, where the amplitude and the delays for the CIR estimated perform a multidimensional search to discover the position of the emitter.

## 4. Experimental Setup

A validation procedure to test the method was performed using real measurements in an outdoor scenario, where a TDOA localization system with four sensors was deployed on the campus of the Technical University of Ilmenau. A UAV acting as an emitter was placed first in LOS situation and then was moved to an NLOS position. The UAV precise coordinates were recorded using a GPS tracker to work as a “ground truth” to check the error in estimation. The scenario setup, with the sensor position, the emitter tracks, and the building information was simulated using ray tracing.

**Figure 15:**
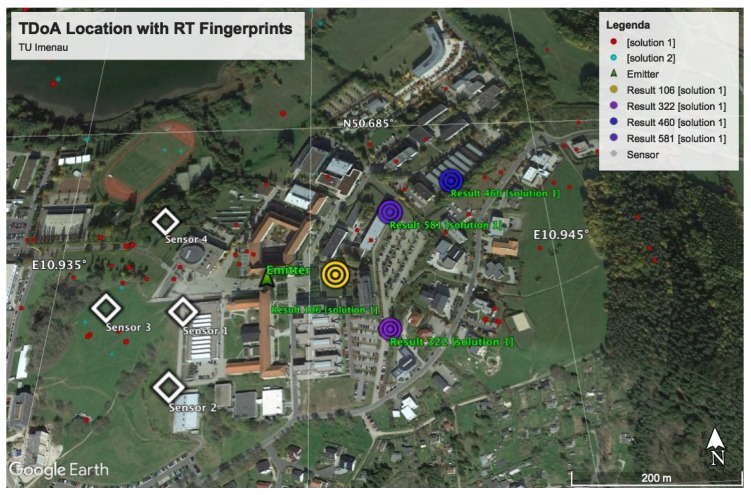
Location error caused by multipath situation in TDOA location.

The scenario with the location performance of TDOA in NLOS is displayed in Figure 11 and Figure 15. In Figure 15, some of the calibration and reference points are displayed in blue circles, the real emitter position is displayed using concentric yellow circles, and the TDOA sensors are indicated with diamonds marks. Figure 11 presents the emitter position in perspective as well as the NLOS situation and the real scenario prepared for ray-tracing simulation.

To build the simulation dataset, first the ray-tracing output was used to load neural network with eight neurons, and then several recorded signals from a known position called “calibration” points were feed into the NN using the CIR estimation and the known ray-tracing fingerprint to refine the dataset. A machine-learning engine was fed the weighting and performed the refinement in the NN using directly the estimated and measured CIR in RT.

A new dataset for machine learning framework was built with the anchor multipath estimation, giving a more precise characterization of the radio channel. Then, the CIR estimated from the target with up to five rays (delay and power) was used to search in the fingerprint database the position of the emitter.

**Figure 16:**
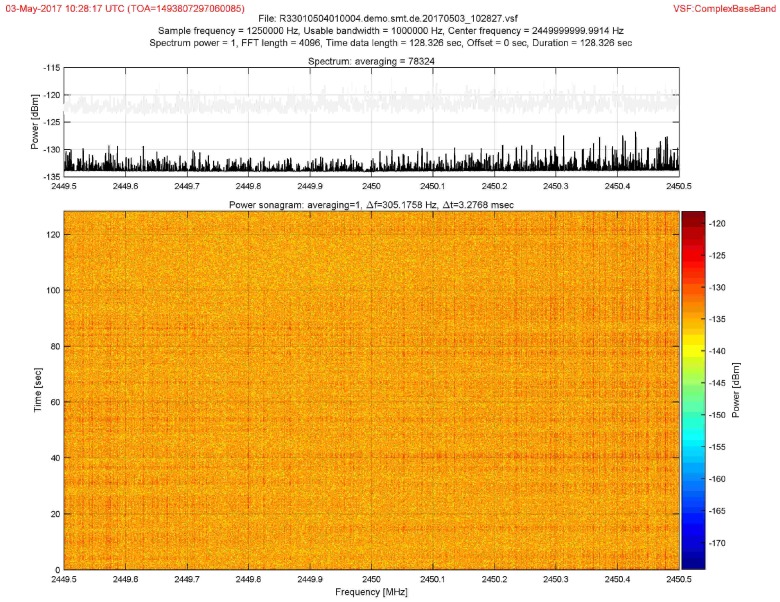
Complex base band signal measured from UAV.

The complex baseband signal recorded by each sensor was processed off-line using MUSIC in the time domain [3] to extract the Channel Impulse Response (CIR) of the calibration points. Finally, the machine-learning engine was fed the ray-tracing dataset, and the hyper-parameters were refined using the α,τ patterns from the CIR of the calibration points. Figure 16 shows the CBB signal from the UAV in a calibration point received by Sensor 1.

The signal transmitted by the UAV was a video-link with the central frequency of 2.4 GHz, with 4 MHz of bandwidth and 20 dB mW (EIRP). The signal was detected, filtered, and down-converted, with the Complex Base Band (CBB) recorded at each sensor node using 1 MHz bandwidth, sampled at 1.25 MHz. A Farrow filter was applied to up-sample the CBB signal, increasing the precision in localization, as described in [33].

The UAV was positioned in an NLOS situation, where there were buildings causing reflection (multipath), giving a disturbance in TDOA measurements.

### 4.1. Multipath Fingerprint Dataset Preprocessing

The raw dataset produced showed strong nonlinearity, and some operations into the raw database were needed to improve the precision. Results from first tries in the classification procedure have not shown further improvement compared to the standard position estimation. Actually, in some cases, the error was even more significant.

**Figure 17:**
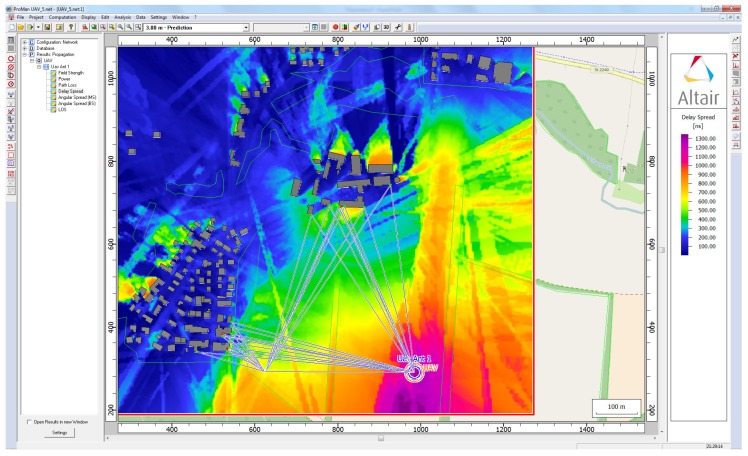
Ray-tracing fingerprints dataset.

The simulation domain has a lot of outlier information regarding possible target position. In this sense, the emitter inside buildings was extracted out from the raw dataset; later analysis revealed that adapting the raw data (delays and power) obtained from simulation to machine learning framework needed to be improved because it gave poor results on training and low accuracy as compared to the expected one. It was necessary to split the simulation domain into slices and normalize the values of the power and delays. Figure 17 shows the multipath pattern loaded in machine learning engine produced by ray-trace simulation.

**Figure 18:**
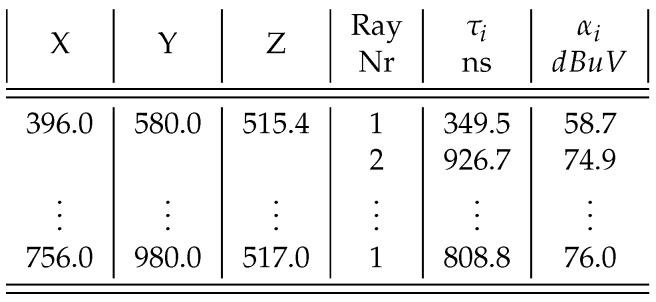
RT simulation output.

As shown in Figure 18, the values of $\tau_{i}$ gives the path delay, the values, $\alpha_{i}$, the field strength of the *i*th path the signal received. The parameters wx, wy, and wz are weights of delay in the hypothesis, and FSWx, FSWy, and FSWz are weights of field strength parameter in the direction of X, Y and Z axes, respectively. The last three parameters, kx, ky, and kz, are intercepts of liner hypothesis in the directions of X, Y, and Z, respectively. In total, the are nine parameters to tune.

Following the steps are given by Abadi et al. [46], the selected raw dataset needed to be labeled, formatted, cleaned and sampled. The labeling was done by taking the data and organizing the amplitude and delay patterns to each point in the simulation domain. The data cleaning procedure was the operation to check if some data need to be removed or fixed, which often happens when some points have no ray information from the simulation. These points need to be removed or zero padded. The sampling was done to make the dataset representative for some problem; in the case of ray-tracing output, the only way to have a re-sampled dataset is either performing the simulation with different grid discretization sizes or taking several calibrations points information to estimate the CIR over and over to refine the dataset. First, the simulation domain was clustered and split into slices, and then the values of the time delays and the amplitude were normalized.

**Figure 19:**
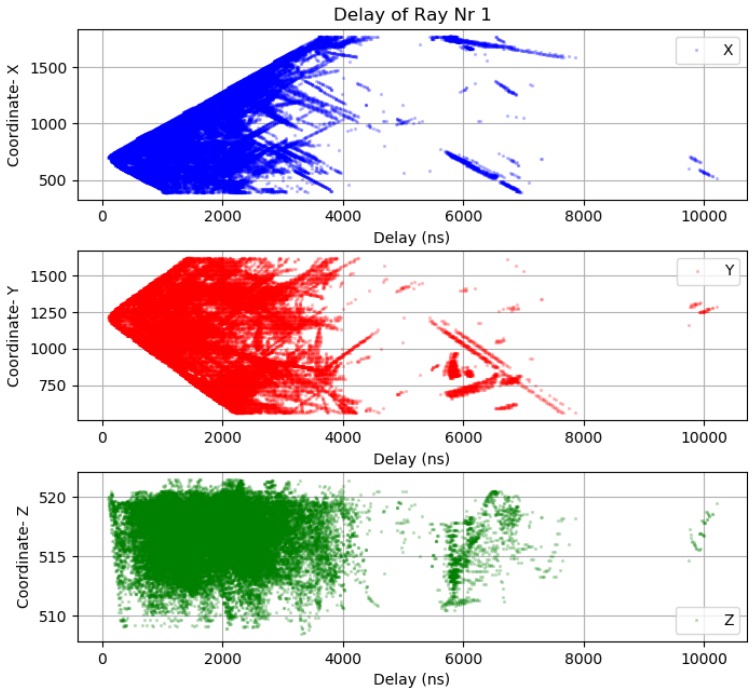
Ray-tracing delay fingerprint Sensor 1.

**Figure 20:**
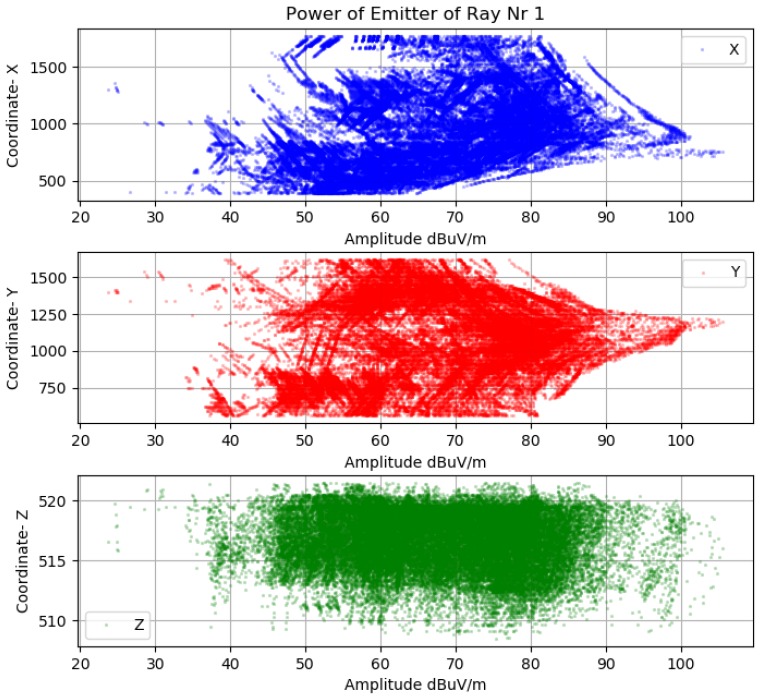
Ray-tracing amplitude fingerprint Sensor 1.

**Figure 21:**
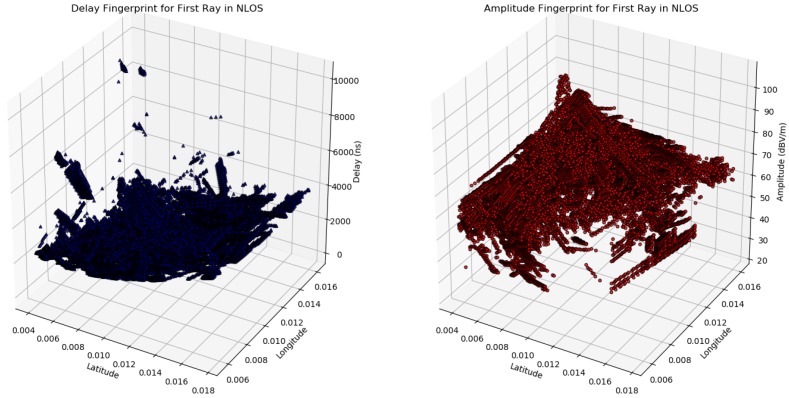
Multipath fingerprint of Ray 1 in Sensor 1.

Before making further assumptions, the dataset was tested to see whether the relationship between input and output is linear, and then a noise remove procedure to extract the outliers in the measurements was performed. The collinearity was removed by calculating pairwise correlations for input data and removing the most correlated. Once the data were prepared, several data analysis and visualization techniques were used as a basis for experimentation to come up with a suitable regression model. The multipath dataset preprocessed to be used in machine learning engine is displayed in Figure 19, Figure 20 and Figure 21.

Then, the CIR estimated from the target with up to five rays (delay and power) was used to search in the fingerprint database for an estimated emitter position. The dataset obtained is shown in Figure 18.

One thousand “training” sequences from the calibration points were necessary to train the model in such a way that the method starts to give an improvement in the position estimation.

### 4.2. Hypothesis and Loss Function

A typical machine regression algorithm [47] has two components: hypothesis (i.e., model) and loss function (i.e., cost function). From the simulated dataset, we have three dependent (emitter position [$X_{e},Y_{e},X_{e}$]) and twenty independent variables, $\alpha_{i},\tau_{i},\;,i=1,\ldots ,5$.

From the data structure of the problem, using the framework from [47] connecting dependent variables (path delay and field strength for up to five rays at each sensor position) and independent variables (geolocation co-ordinates X, Y, Z), we chose following hypothesis for our machine to train. For each ray taken from ray-tracing simulation, the following procedure was performed for $X_{e},Y_{e},Z_{e}$.

(30) $$\begin{array}{cc} X_{e} & =\{w_{x}*\left(\tau_{1}^{2}+\tau_{2}^{2}+\tau_{3}^{2}+\tau_{4}^{2}+\tau_{5}^{2}\right)\\ & +FSWx*\left[\left(\alpha_{1}^{2}-\alpha_{1}\right)+\left(\alpha_{2}^{2}-\alpha_{2}\right)+\left(\alpha_{3}^{2}-\alpha_{3}\right)+\left(\alpha_{4}^{2}-\alpha_{4}\right)+\left(\alpha_{5}^{2}-\alpha_{5}\right)\right]{\}}^{\;1/2}+k_{x} \end{array}$$

(31) $$\begin{array}{cc} Y_{e} & =\{w_{y}*\left(\tau_{1}^{2}+\tau_{2}^{2}+\tau_{3}^{2}+\tau_{4}^{2}+\tau_{5}^{2}\right)\\ & +FSWy*\left[\left(\alpha_{1}^{2}-\alpha_{1}\right)+\left(\alpha_{2}^{2}-\alpha_{2}\right)+\left(\alpha_{3}^{2}-\alpha_{3}\right)+\left(\alpha_{4}^{2}-\alpha_{4}\right)+\left(\alpha_{5}^{2}-\alpha_{5}\right)\right]{\}}^{\;1/2}+k_{y} \end{array}$$

(32) $$\begin{array}{cc} Z_{e} & =\{w_{z}*\left(\tau_{1}^{2}+\tau_{2}^{2}+\tau_{3}^{2}+\tau_{4}^{2}+\tau_{5}^{2}\right)\\ & +FSWz*\left[\left(\alpha_{1}^{2}-\alpha_{1}\right)+\left(\alpha_{2}^{2}-\alpha_{2}\right)+\left(\alpha_{3}^{2}-\alpha_{3}\right)+\left(\alpha_{4}^{2}-\alpha_{4}\right)+\left(\alpha_{5}^{2}-\alpha_{5}\right)\right]{\}}^{\;1/2}+k_{z} \end{array}$$

where $w_{x},w_{y},w_{z}$, and $k_{x},k_{y},k_{z}$ are the adjustment parameters refined by the multipath information given by the calibration emitters. There are nine parameters to tune; in the experimental phase of this work, we investigated seven different models, changing up to thirty parameters. None of those permutations were statistically better or worse, permitting random noise of order during the training phase.

Here, $\tau_{i}$ measures path delay while $\alpha_{i}$ measures field strength of the *i*th path the signal received. Parameters $w_{x},w_{y},w_{z}$ are weights of delay in the hypothesis and *FSWx*, *FSWy*, and *FSWz* are weights of field strength parameter in the direction of X, Y and Z axes, respectively. The last three parameters, $k_{x},k_{y},k_{z}$ are intercepts of linear hypothesis in the directions of X, Y, and Z, respectively. In total, there are nine parameters to tune. In the experimental phase of this work, we investigated seven different models of up to thirty parameters. None of those permutation were statistically better or worse, permitting random noise of order during the training phase.

A loss function or cost function is a function that maps an input data values of one or more variables onto a real number representing loss/cost with associated event. While there are numerous regression loss functions, we focused on the mean squares (MS) function.

The mean square error of the measurements was taken from the difference between predicted geolocation and that measured during simulation. The next three expressions are mean square error functions of coordinates X, Y, and Z, respectively. The fourth expression is the loss function that represents the error of the first three mean square error functions. The loss function is used during training to minimize overall loss as a mean square error.

(33) $$\begin{array}{ccc} MSE_{x}=\frac{1}{n}\sum_{i=1}^{n}{\left(X_{pred}-X_{sim}\right)}^{2} & MSE_{y}=\frac{1}{n}\sum_{i=1}^{n}{\left(Y_{pred}-Y_{sim}\right)}^{2} & MSE_{z}=\frac{1}{n}\sum_{i=1}^{n}{\left(Z_{pred}-Z_{sim}\right)}^{2}\\ & Loss=\sqrt[{2}]{MSE_{x}+MSE_{y}+MSE_{z}\;} & \end{array}$$

Our simulated dataset consists of over 46,000 samples. We noticed the range of the dataset values was orders of magnitude. In particular, delay values were from 100 to 11,000. We normalized our dataset, using well-known z-scores [48].

(34) $$z_{score}=\frac{data-\mu }{\sigma }$$

μ,σ are the mean and standard deviation of each variable in the dataset. Based on [49], randomization in the dataset was performed to improve the efficiency of our training and to reflect field environment. The total number of training samples was over 37,000.

**Figure 22:**
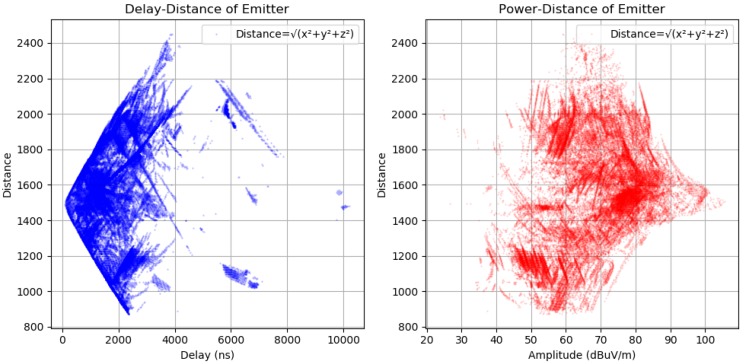
Testing the dataset with delay–distance and power–distance mapping.

As shown in Figure 22, the dataset was analyzed regarding delay–distance and power–distance relationships to extract the features for the machine-learning engine.

### 4.3. Training and Model Generation

The graph was created with the hypotheses and loss function using eight neurons for every coordinate (X, Y, Z) of geolocation with a total of 24 neurons. Once the compute graph was ready, an optimization algorithm gradient descent was applied to optimize the loss function described in the previous section. The so-called "learning rate”, defined in [42],was fixed at 0.1, as a hyper-parameter to improve accuracy. Resulting learning network was trained for 1000 epochs. With the "trained” dataset model, all nine model parameters were saved. These parameters were used in creating a stand-alone model ready for in-house testing and subsequent deployment in the field. Testing was performed using this model with 20% of the dataset set aside during the data conditioning phase.

### 4.4. Results

An accuracy of 2% was achieved, representing the error in the result of the application of the method using 1000 epochs to training and optimize the loss function. The training dataset error mean =1.303%. The same model was used on the test dataset, and accuracy was found within the same range and the test dataset error mean =1.322%

**Figure 23:**
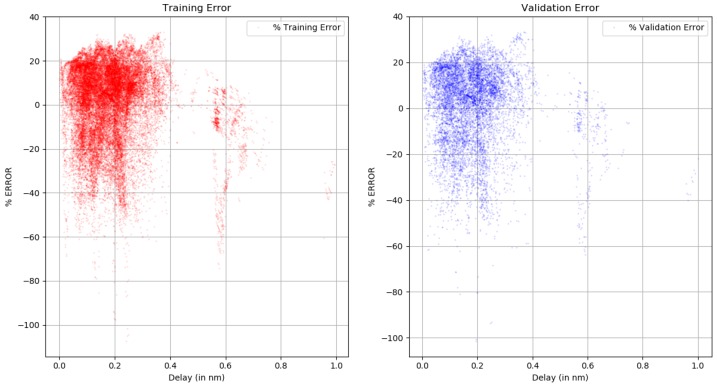
Error analysis of training data and test dataset.

The training set and the test set are displayed in Figure 23. The trends in both these charts reflect that data were randomized in a statistically significant manner to show that model can generalize well over a large dataset of over 46,000 samples. The localization procedure is not straightforward to analyze; the machine-learning engine and ray-tracing simulation are two tools that only give reasonable results depending on the obstacle representation quality in the geographic database. Machine learning can improve the multipath fingerprint generated by ray tracing, but it also depends on the capability of processing the channel impulse estimation and getting an appropriate multipath characterization of the calibration point.

The method was improved to avoid dealing with ambiguities in the position estimation, once only the time differences that intercept the three subsets ($\tau_{21,\tau_{31},\tau_{41}}$), were considered in the calculations. The scenario and geographic features were included in the position processing estimation to improve the emitter localization precision.

Table 2 summarizes the performance of the proposed method. The values for the mean and variance in time-differences $\tau_{21},tau_{31},tau_{41}$ given by the TDOA system measurements, the ground-truth and the proposed method show the improvement in the error reduction. Sensor 1 is close to the emitter, and Sensor 4 is in LOS situation, thus the TDOA measurement errors are smaller than those for other sensors, which is why the reduction in the error of the time-difference $tau_{41}$ was a little bit lower than the improvement in $\tau_{21},tau_{31}$.

**Figure 24:**
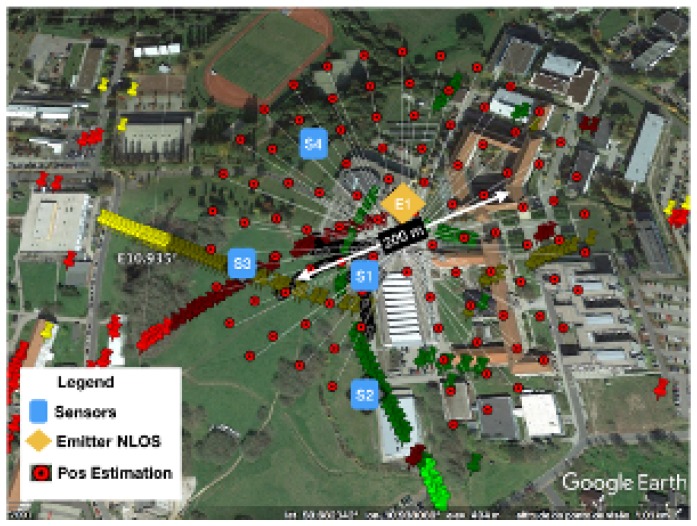
Position estimation error in NLOS.

**Figure 25:**
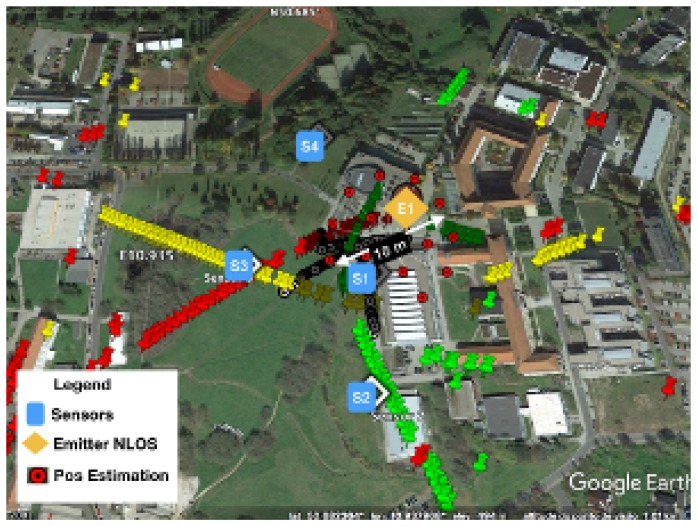
Final position estimation error with machine learning RT.

The performance is also displayed in Figure 24 and Figure 25. Figure 24 shows the dispersion of position estimation within 200 m of the real position, which means that the error in the time-differences estimation caused by the NLOS condition affects the final results of the system. The improvement in the time-differences estimation in Table 2 is shown in Figure 25, where the joint estimation using the ray tracing multipath fingerprints has improved the performance, and the dispersion was reduced to within 18 m of the real emitter position.

As can be seen, several estimates are recursively done taking more and more samples from the signal and the calibration signals. The “reinforcement” in the learning process is done by an improvement in the hyper-parameters used, the position is estimated by using a joint estimation procedure enhancing the performance of the TDOA system in NLOS situation.

The improvement in emitter location is achieved with ray tracing and machine learning using the dataset from calibration emitters in a real scenario. It binds together the RSS fingerprint approach, with five rays and four different base stations.

## 5. Conclusions

This paper proposes a kernel-based machine learning localization scheme based on TDOA fingerprinting. It describes how to use ML for refining the Multipath Fingerprint generated by ray tracing simulation. Despite the bias and imprecision of the scenario description, the main obstacles that affect the signal in a given frequency are the buildings, taken into account by ray trace simulation.

An enhancement of the ray tracing fingerprint approach was proposed to add the time differences database to the classical RSS approach. Despite the imprecision in building description, the uniform theory of diffraction implemented in the simulation can give the effects in the propagation paths that influence the TDOA system.

The results, using real measurements set, show that geo-information is a field to be further explored because it can further improve radio frequency system performance. In this sense, the scenario features, summed up in the cartographic maps, can give the signal processing an extra dimension of work.

We also show that our method is very insensitive and measurement errors from the reference nodes that are randomly chosen form emitter tracks in the area of interests. These features make our approach very appealing for practical applications in NLOS propagation environments. The results applying this method in a real scenario show that our approach can derive more accurate localization than RSSI fingerprinting procedures and the previously published TDOA fingerprints approach. We also show that the method can deal with measurement errors by continuously improving the estimation based on the measurements available in the area of interests. These features make our approach very appealing for practical applications in NLOS propagation environments.

The effect in localization procedure is not straightforward to analyze; the ML engine and ray tracing are two tools that only give reasonable results depending on the quality of obstacle representations in a geographic database. The ML can improve the multipath fingerprint generated by ray tracing, but it also depends on the capability of processing the channel impulse estimation and getting an appropriate multipath characterization of the calibration point.

The real signal in the environment needs to be up-sampled and filtered to have an NLOS description that matches with the ray tracing simulations. However, the approach showed that further analysis could be done to improve the idea of using NLOS reflection information; embedded into RT simulation, it can handle NLOS environments as well as LOS propagation.

For next steps in the research, the authors would like to consider the use of more features, either signals or scenarios, trying to record more multipath information and patterns to build a data fusion engine with the cartographic database and signal processing. The authors also plan to use optimization tools for Sensor Management and to apply the machine learning approach to deal with the rough information produced by multipath reflection in the TDOA system deployment. 

## Figures and Tables

**Table 1:** Pseudo-code for machine learning NN fingerprints.

Algorithm: TDOA with RT Fingerprints.	
1:	**Begin:**
2:	**From a deployed TDOA System:**
	· Record the signal, extract the CBB;
	· Extract a “windows” in time domain of the CBB signal;
	· Using the sample, extract TDOA vector $\left[\tau_{21},\tau_{31},\tau_{41}\right]$;
	· Estimate the emitter position.
3:	**Perform Ray Tracing Simulation of the Scenario with:**
	· TDOA Sensor Positions,
	· Emitter position vector x,
	· Buildings descriptions.
4:	**With RT output collect Amplitude ($\alpha_{i}$) and Delay ($\tau_{i}$)**
5:	**Build a Neural Network (NN) with:**
	· 40 Input: 5 ($\alpha_{i},\tau_{i}$) for each TDOA Sensor;
	· 8 Neurons in the hidden layer;
	· weights and hyper-parameter for “Supervised Learning”.
	· 3 Outputs: Emitter Position ${\left[X_{e},Y_{e},Z_{e}\right]}^{T}$.
6:	**Refine the NN:**
	· Record from the Calibration;
	· Perform CIR estimation to extract ($\alpha_{i},\tau_{i}$) from calibration;
	· Perform hyper-parameters refinement;
	· Adjust the dataset of Machine Learning Engine.
7:	**Perform the CIR of the emitter:**
	· Refine the NN adjustments from calibration.
8:	**Perform the Position Estimation**
	**Apply K-nearest neighbor(KNN) method and NN refined**.
9	**Repeat the algorithm from step 4**
	**with a new integration windows**
	**untill the end of the recording signal.**
10:	**Perform the joint position estimation**
	**with $P\left(\mu_{\mathrm{TDOA}},\sigma_{\mathrm{TDOA}}\right)\cap P\left(\mu_{\mathrm{RT}},\sigma_{\mathrm{RT}}\right)$.**

**Table 2:** Improvement in TDOA position estimation.

TDOA	Ground Truth GPS	Measurements		Proposed Method (μs)	Error Reduction (Improment)
		Mean (μs)	Variance (μs)				
$\tau_{21}$	0.333	0.114	0.0341	0.344	94%
$\tau_{31}$	0.27	1.76	0.0303	0.266	96%
$\tau_{41}$	0.266	0.15	0.0157	0.238	79%

## Abbreviations

The following abbreviations are used in this manuscript:

AOA	Angle of Arrival
CBB	Complex Baseband
CIR	Channel Impulse Response
CRB	Cramer-Rao Bound
DMC	Dense Multipath Components
DOA	Direction of Arrival
EKF	Extended Kalman Filter
FDOA	Frequency Difference of Arrival
FIM	Fisher Information Matrix
LS	Least Squares
LLS	Linear Least Squares
KF	Kalman Filter
ML	Maximum Likelihood
NLOS	Non-Line-of-sight
NLS	Nonlinear Least Squares
TDOA	Time-difference of Arrival
RSS	Received Signal Strength
RX	Receiver
SC	Specular Components
SDP	Semidefinite Programming
SVM	Support Vector Machine
TMA	Target Motion Analysis
TOA	Time of Arrival
TTW	Through-the-wall
TX	Transmitter
WLLS	Weighted Linear Least Squares
